# Epidemiology and demographic trends of sarcoidosis in southern West Bank, Palestine: a retrospective study (2019–2024)

**DOI:** 10.1186/s12889-026-27878-0

**Published:** 2026-05-22

**Authors:** Mohanad Saleh, Tasneem Mustafa Almashaiekh, Rahaf Wasfi Thabaineh, Rawda Qasrawi, Ikram Aldarabee, Rana Wridat, Motaz Natsheh, Yousef Atef Abu Asbeh

**Affiliations:** 1 Department of Medicine, Palestine Polytechnic University, Hebron, Palestine; 2 Department of Medicine, Dura General Hospital, Hebron, Palestine; 3 College of Medicine and Health Sciences, Palestine Polytechnic University, Hebron, Palestine; 4 Palestine Red Crescent Society (PRCS) Hospital, Hebron, Palestine; 5 Department of Pathology, Al Ahli Hospital, Hebron, Palestine; 6 Thoracic Surgery Unit, Al Ahli Hospital, Hebron, Palestine

**Keywords:** Sarcoidosis, Epidemiology, Incidence, Prevalence, Hebron, Bethlehem, Retrospective study

## Abstract

**Background:**

Sarcoidosis is a systemic granulomatous disease of unknown etiology that most commonly affects the lungs and hilar lymph nodes. Although it has been reported worldwide, its epidemiology and clinical characteristics in Palestine remain unclear. This study aimed to estimate the incidence, prevalence, and sociodemographic characteristics of sarcoidosis diagnosed between 2019 and 2024 in the southern West Bank of Palestine (Hebron and Bethlehem).

**Methods:**

A retrospective observational study was conducted using medical records from Al-Ahli Hospital and affiliated pathology laboratories. Data collected included annual incidence and prevalence rates, age at diagnosis, sex, diagnostic method, biopsy site, smoking status, and comorbidities. Descriptive statistics were calculated, and inferential analyses included chi-square tests for categorical variables and independent-samples t-tests for continuous variables. Statistical analyses were performed using Statistical Package for the Social Sciences (SPSS) version 27, with $p < 0.05$ considered statistically significant.

**Results:**

A total of 62 patients were diagnosed with sarcoidosis during the study period. The annual incidence increased from 0.41 to 1.92 per 100,000 population between 2019 and 2024, while prevalence increased from 0.41 to 5.67 per 100,000. The mean age at the time of diagnosis was $48.5 \pm 16.4$ years, and most cases occurred in individuals aged $\ge$ 36 years. Females accounted for 61.3% of cases. Diagnosis was histologically confirmed in 83.9% of patients, most commonly from thoracic lymph nodes (46.8%). Statistically significant gender differences were observed in smoking status ($p = 0.0001$) and hypothyroidism ($p = 0.023$), while no statistically significant difference were observed in mean age at diagnosis between males and females ($p = 0.47$). Trend analysis showed a statistically significant increase in reported incidence over time.

**Conclusion:**

Sarcoidosis incidence and prevalence increased in the southern West Bank, particularly after 2022. The disease was observed more frequently among middle-aged adults and females. The observed increase likely reflects improvements in diagnostic capacity and greater clinical awareness; however, a true rise in disease occurrence cannot be excluded. These findings highlight the need for improved diagnostic access, clinician education, and the establishment of a national sarcoidosis registry to improve disease surveillance and patient outcomes in Palestine.

**Trial registration:**

Not applicable. This study did not involve a healthcare intervention in human participants.

**Supplementary Information:**

The online version contains supplementary material available at 10.1186/s12889-026-27878-0.

## Introduction

Sarcoidosis is a chronic immune-mediated granulomatous disease characterized by the formation of noncaseating granulomas in affected tissues. Although it can affect almost any organ system, pulmonary involvement is the most common manifestation, usually affecting the lungs and intrathoracic lymph nodes, followed by ocular, cutaneous and neurological involvement [1, 2]. The underlying pathogenesis remains poorly understood but is hypothesized to involve an exaggerated immune response to unidentified environmental,infectious or occupational antigens in genetically predisposed individuals [2].

In 2021, the global burden of pulmonary sarcoidosis was estimated at approximately 43 million cases worldwide, with the highest prevalence reported in Northern Europe and North America [3]. The disease typically presents in adults aged 35 to 55 years and demonstrates a slight female predominance [4]. Recent epidemiological findings suggest that women are frequently diagnosed up to a decade later than men, and females constitute approximately 60 to 70% of cases in multiple cohorts, a pattern particularly notable among African American populations [5]. These disparities suggest potential sex-based and ethnic influences on disease presentation.

The clinical presentation of sarcoidosis is highly variable, depending on the organ involved. Pulmonary manifestations are the most common, occurring in up to 90% of cases and typically present with cough, dyspnea, and chest discomfort. Systemic symptoms may also be reported that include fever, fatigue, weight loss, and night sweats. Extrapulmonary manifestations are also common, including uveitis,cutaneous lesions,and cardiac arrhythmias [6, 7].

Diagnosis relies on clinical evaluation, radiographic imaging, and histopathological confirmation. Chest radiography and high-resolution CT (computed tomography) are valuable tools, but definitive diagnosis requires tissue biopsy demonstrating non-caseating granulomas in the absence of alternative causes [8]. In Palestine, limited access to advanced diagnostic methods and histopathology contributes to frequent diagnostic delays and potential misdiagnosis with tuberculosis or lymphoma, which share similar clinical and radiographic features [8, 9]

While spontaneous remission may occur in many patients,treatment is required in cases with severe, progressive symptoms or organ-threatening disease. Corticosteroids are the first-line therapy for sarcoidosis,while immunosuppressive agents such as methotrexate or tumor necrosis factor-alpha (TNF-$\alpha$) inhibitors are used for severe or refractory cases [10–12].

Beyond its multisystem involvement,sarcoidosis is increasingly associated with comorbid chronic conditions. These include autoimmune thyroid disorders, most notably Hashimoto’s thyroiditis, as well as type 2 diabetes mellitus and hypertension. Such complications may arise from chronic inflammation or as adverse effects of long-term corticosteroid use. In regions already burdened by a high prevalence of noncommunicable diseases,these associations highlight the importance of integrated and multidisciplinary care strategies [13–15].

Despite increasing global awareness, epidemiological data from the Middle East—and particularly Palestine—remain limited. Most regional studies focus broadly on interstitial lung diseases without distinguishing sarcoidosis-specific patterns. Consequently, there are currently no published estimates of incidence, prevalence, or clinical characteristics of sarcoidosis in the Palestinian population [9].Contributing factors may include limited medical records, insufficient diagnostic tools, and low awareness among healthcare personnel. Environmental exposures, such as dust and infectious agents, may influence disease occurrence, while sex and geographic factors may be associated with differences in presentation and outcomes [3, 5, 9].

To address this gap, this study provides the first regional epidemiological evaluation of sarcoidosis in the West Bank of Palestine, focusing on cases diagnosed in the West Bank between 2019 and 2024. We examine the incidence, prevalence, and clinical and demographic characteristics of affected patients. These findings aim to raise local awareness, guide diagnostic and management strategies, and contribute to the regional and international epidemiological understanding of sarcoidosis.

## Methods

### Study design

The study was designed as a retrospective observational study evaluating the epidemiological and clinical characteristics of sarcoidosis in the southern West Bank of Palestine, covering the period between January 2019 and December 2024. Data were obtained from the electronic medical records of Al-Ahli Hospital and affiliated pathology laboratories in the Hebron and Bethlehem governorates.

All suspected sarcoidosis cases from the southern West Bank are referred to Al-Ahli Hospital, which serves as the only regional center where histopathological confirmation is routinely performed. Therefore, the hospital’s records are considered representative of confirmed sarcoidosis cases in this region.

Al-Ahli Hospital functions as the main tertiary referral center for the histopathological diagnosis of sarcoidosis in the southern West Bank. During the study period (2019–2024), it was the only facility in the region where specialized pathological services performed and interpreted tissue biopsies routinely for suspected sarcoidosis. As a result, patients from both Hebron and Bethlehem governorates who required diagnostic confirmation were systematically referred to this center, regardless of their initial clinical presentation site, including public hospitals, private clinics, pulmonology practices, and outpatient services.

Nevertheless, a limited number of cases diagnosed clinically without histopathological confirmation or patients referred outside the region may not have been captured in the hospital records.

Ethical approval was obtained from the Institutional Review Board and Ethics Committee of the Medical School, Palestine Polytechnic University (Approval No: EA/2025/80). The Ethics Committee also approved a waiver of informed consent, as the study was retrospective, used anonymized medical records, and involved no direct patient contact.

### Study population

Patients were eligible for inclusion if a diagnosis of sarcoidosis was confirmed based clinical, radiological, or histopathological criteria during the defined study period.

#### Inclusion criteria

 

- Diagnosis of sarcoidosis confirmed by histopathological evidence of noncaseating granulomas in pulmonary or extrapulmonary tissues, or by compatible clinical and radiological features when biopsy was not feasible.
- Diagnosis made between January 2019 and December 2024.
- Residency in the southern West Bank of Palestine (Hebron or Bethlehem governorates).

#### Exclusion criteria

 

- Medical records lacking sufficient diagnostic or demographic detail.
- Cases with uncertain or provisional diagnoses of sarcoidosis.

A total of 62 patients met the inclusion criteria and were included, 44 had available past medical history, and 45 had data on smoking status.

The determination of the case relied on centralized diagnostic referral pathways, allowing suspected cases from the Hebron and Bethlehem governorates to be referred to Al-Ahli Hospital for definitive diagnostic confirmation. To enhance completeness, hospital pathology records were cross-checked with affiliated pathology laboratories. Nevertheless, cases that did not undergo biopsy—particularly mild or asymptomatic presentations—may not have been captured.

### Case definition and diagnostic criteria

The diagnosis of sarcoidosis was established based on compatible clinical and radiological findings, supported by histopathological confirmation of noncaseating granulomas when tissue biopsy was performed and after exclusion, and after careful exclusion of alternative causes of granulomatous disease, including tuberculosis or fungal infections. Diagnostic evaluation was conducted according to internationally accepted criteria recommended by the American Thoracic Society, the European Respiratory Society, and the World Association of Sarcoidosis and Other Granulomatous Disorders (ATS/ERS/WASOG).

Histopathological identification of noncaseating granulomas was considered the gold standard. Whenever feasible, tissue biopsy specimens were obtained from affected organs and examined by specialized pathologists at Al-Ahli Hospital. In cases where biopsy was not performed or was not feasible, diagnosis was established based on a combination of characteristic clinical presentation, radiological findings, and specialist clinical judgment after exclusion of other granulomatous diseases.

### Data collection

Data were extracted from patient medical records using a standardized data collection form. The following variables were retrieved and analyzed:

- Demographic characteristics: including age at diagnosis and sex.
- Incidence rates: calculated annually as the number of new sarcoidosis cases per 100,000 population, using official mid-year population estimates from the Palestinian Central Bureau of Statistics (PCBS) for Hebron and Bethlehem governorates between 2019 and 2024
- Diagnostic method: classified as either biopsy-confirmed diagnosis (histopathological evidence of noncaseating granulomas) or clinical diagnosis based on compatible presentation and imaging findings.
- Biopsy site: documented when applicable, including thoracic lymph nodes, extrathoracic lymph nodes, skin, and other organ systems.
- Smoking status: classified as smoker or non-smoker, based on available patient history.
- Comorbid conditions: including hypertension, diabetes mellitus, gout, hypothyroidism, congestive heart failure, and chronic kidney disease.

Detailed clinical variables—such as presenting symptoms, radiographic staging (e.g., Scadding classification), and systematic documentation of extrapulmonary involvement were not consistently available in patient records and therefore were not included in the analysis.

Data were coded, anonymized, and securely stored for analysis using SPSS (version 27; IBM Corp., Armonk, NY, USA) and Microsoft Excel, with all personal identifiers removed to ensure confidentiality.

### Statistical analysis

Data from 62 eligible patients were entered, coded, and analyzed using SPSS version 27 (IBM Corp., Armonk, NY, USA) and Microsoft Excel. Descriptive statistics were computed for demographic and clinical characteristics, with continuous variables presented as means ± standard deviation (SD) and categorical variables as frequencies and percentages.

Incidence and prevalence rates were calculated per 100,000 population using official Palestinian Central Bureau of Statistics (PCBS) estimates for the Hebron and Bethlehem governorates (2019–2024) [16, 17]. Ninety-five percent confidence intervals (95% CIs) were calculated using the Poisson distribution appropriate for count data.

Trends in incidence and prevalence over the study period were evaluated using linear regression and the Cochran–Armitage test for trend. Associations between categorical variables (e.g., sex, smoking status, comorbidities) were assessed with the chi-square test or Fisher’s exact test, as appropriate. Differences in mean age between male and female patients were assessed using the independent samples t-test.

The missing data ranged from 27.4% to 29% in key variables, particularly smoking status and history of comorbidities. This missingness reflects the retrospective nature of the study and variability in historical medical record documentation. The pattern of missingness was unlikely to be completely random. However, given the modest sample size and risk of introducing further bias, imputation or sensitivity analyses were not performed. Analyses were therefore conducted using available complete-case data. A *p*-value < 0.05 was considered statistically significant.

## Results

A total of 62 patients were diagnosed with sarcoidosis across the Hebron and Bethlehem governorates districts between 2019 and 2024. The data revealed evolving trends incidence and prevalence, demographic characteristics, diagnostic modalities, and associated comorbidities during the study period.

### Incidence rate analysis (2019–2024)

**Fig. 1:**
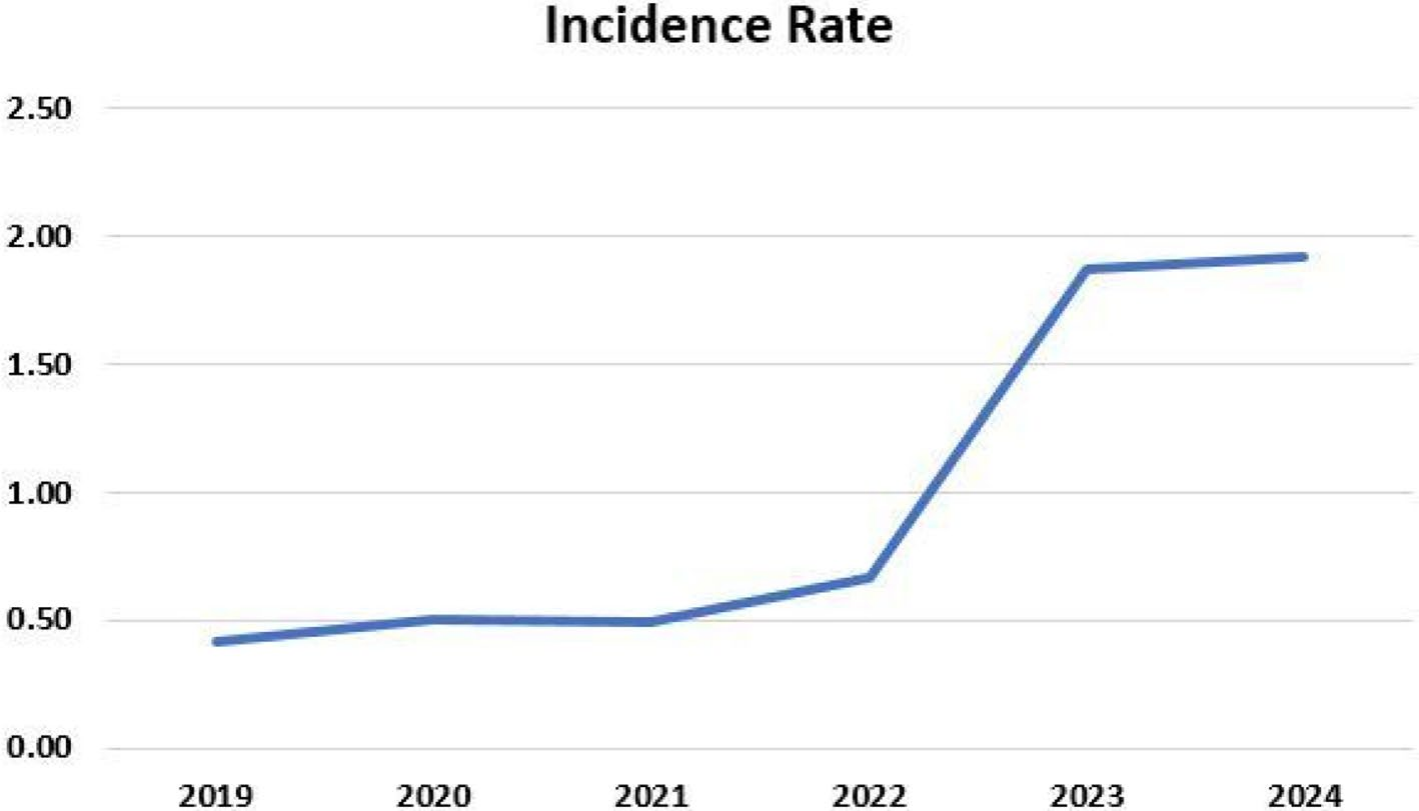
Annual incidence rate of sarcoidosis in Hebron and Bethlehem, 2019–2024. This figure illustrates the annual incidence rate per 100,000 population across the study period. A gradual rise was observed until 2022, followed by a sharp increase in 2023 and 2024, indicating improved disease recognition or reporting

The incidence rate of sarcoidosis in Hebron and Bethlehem demonstrated an overall upward trend during the study period (Table 1, Fig. 1). When population-adjusted incidence was calculated, the rate increased from 0.41 per 100,000 in 2019 to 1.92 per 100,000 in 2024—representing nearly a five-fold rise over six years.

Between 2019 and 2022, incidence rates remained relatively stable, fluctuating between 0.41 and 0.67 per 100,000, which is consistent with the modest annual number of new cases (4–7 per year). This stability is evident in Fig. 1, which shows only minimal year-to-year variation.

A noticeable increase was observed in 2023, when the incidence rate sharply increased to 1.87 per 100,000, driven by the identification of 20 new cases. This elevated rate persisted in 2024 (1.92 per 100,000) with 21 new cases, suggesting that the increase was not limited to a single year.

The mean incidence rate for the entire period was $0.73 \pm 0.76$ (SD) per 100,000 population, indicating considerable variability, primarily attributable to the abrupt increase observed in the final two years. Collectively, these findings suggest a shift from a relatively stable low-incidence pattern to higher detected rates, which may be influenced by improved diagnostic recognition, enhanced reporting accuracy, or other factors, including a potential true increase in disease occurrence.

**Table 1:** Incidence rate of new cases over Hebron and Bethlehem between 2019–2024

Year	Population	New cases	Incidence rate (per 100,000)	Lower 95% CI	Upper 95% CI
2019	968,141	4	0.41	0.11	1.05
2020	992,425	5	0.50	0.16	1.17
2021	1,017,029	5	0.49	0.16	1.14
2022	1,041,912	7	0.67	0.27	1.38
2023	1,067,139	20	1.87	1.15	2.91
2024	1,092,658	21	1.92	1.19	3.00

(Mean =.73, Standard Deviation =.76) per 100,000, $n = 62$

Annual incidence rates with corresponding 95% Poisson confidence intervals are provided in Supplementary Fig. S1.

### Trend analysis for incidence rates

Trend analysis confirmed a significant increase in incidence between 2019 and 2024 was statistically significant (Table 2). Linear regression analysis demonstrated a significant positive year coefficient ($\beta = 0.3383, p = 0.0197$), indicating a meaningful annual increase in incidence rates. The model explained approximately 78% of the observed variance ($R^{2} = 0.7801$). The F statistic (14.19; $p = 0.01965$) confirmed overall model significance.

Furthermore, the Cochran–Armitage trend test yielded a highly significant chi-square statistic ($\chi ^{2} = 21.032, p < 0.001$), reinforcing the presence of a strong increasing temporal trend.

**Table 2:** Trend test for incidence rates (2019–2024) of cases in Hebron and Bethlehem

Test	Statistic	Value	DF	*P*-value	Interpretation
Linear regression	Intercept Estimate	−682.87		0.0197	Significant intercept
	Year Coefficient Estimate	0.3383		0.0197	Significant positive trend
	Residual Std. Error	0.3757	4	1.38	
	Multiple R-squared	0.7801		2.02	78% variance explained
	F-statistic	14.19	1 and 4	0.01965	Overall model significant
Cochran–Armitage Test	Chi-squared Statistic	21.032	1	0.000	Highly significant increasing trend

### Prevalence rate analysis (2019–2024)

**Fig. 2:**
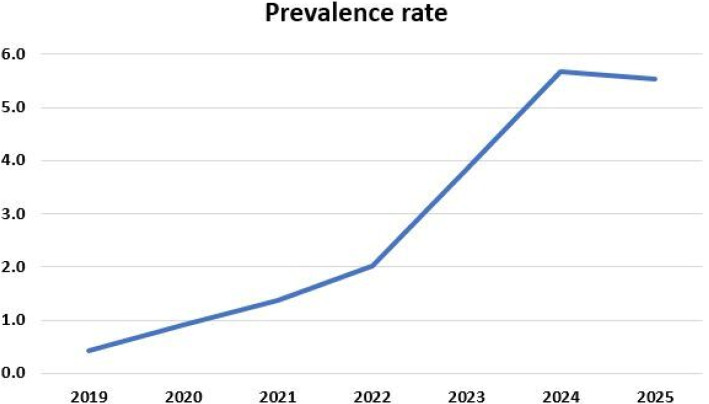
Annual prevalence rate of sarcoidosis in Hebron and Bethlehem, 2019–2024. This figure depicts the cumulative prevalence of sarcoidosis per 100,000 populations, showing a steady increase over time, with a marked acceleration between 2022 and 2024

Prevalence data—combining cumulative diagnosed cases and annual population estimates—demonstrated a persistent and substantial rise in sarcoidosis burden across Hebron and Bethlehem (Fig. 2, Table 3). Prevalence increased from 0.41 per 100,000 in 2019 to 5.67 per 100,000 in 2024—representing more than a 13-fold increase.

This rise closely reflects the accumulation of documented cases, which grew from 4 cases in 2019 to 62 cases in 2024. The line graph (Fig. 2) illustrates a gradual incline from 2019 to 2022, followed by a sharp increase in 2023 when prevalence reached 3.84 per 100,000, and continued to increase in 2024.

**Table 3:** Prevalence rate of cases over Hebron and Bethlehem between 2019–2024

Year	Population	New cases	Cumulative cases	Prevalence rate (per 100,000)
2019	968,141	4	4	0.41
2020	992,425	5	9	0.91
2021	1,017,029	5	14	1.38
2022	1,041,912	7	21	2.02
2023	1,067,139	20	41	3.84
2024	1,092,658	21	62	5.67

Mean = 3.15, Standard Deviation = 2.22 per 100,000, $n = 62$

Annual prevalence rate of sarcoidosis per 100,000 population across the study period, accompanied by 95% confidence intervals are provided in Supplementary Fig. S2.

### Demographic characteristics

The mean age at diagnosis was 48.5 ± 16.4 years, with 80.6% of patients were aged 36 years or older. Specifically, 24.2% were between 48 and 59 years, and 30.6% were aged 60 years or older—the latter comprising the largest single age group. Only 19.4% of patients were under 36 years of age.

Females constituted 61.3% ($n = 38$) of the study population, while males accounted for 38.7% ($n = 24$), resulting in a female-to-male ratio of approximately 1.6:1 (Table 4).

### Diagnostic methods and biopsy sites

A biopsy was performed in 83.9% ($n = 52$) of cases, confirming the diagnosis histopathologically through the identification of noncaseating granulomas. The remaining 16.1% ($n = 10$) were diagnosed based on clinical and radiological features without biopsy. Among the 52 patients who underwent biopsy, various techniques were employed depending on the anatomical site and clinical judgment.These included transbronchial biopsy, fine-needle aspiration, excisional lymph node biopsy, core needle biopsy, Tru-cut biopsy, punch biopsy for cutaneous lesions, and, in selected cases, surgical biopsy via Video-Assisted Thoracoscopic Surgery (VATS). The thoracic lymph nodes were the most common biopsy site (46.8%, $n = 29$), followed by extrathoracic lymph nodes (25.8%, $n=16$), skin (6.5%,$n = 4$), and other anatomical locations (4.8%,$n=3$) (Table 4).

**Table 4:** Distribution of socio-demographics among patients ($N=62$)

Socio-demographics	n	%
Age		
(M ± SD)	48.48 ± 16.4111	
12–23 years	6	9.7
24–35 years	6	9.7
36–47 years	16	25.8
48–59 years	15	24.2
60 years and above	19	30.6
Gender		
Male	24	38.7
Female	38	61.3
Diagnosis method		
Clinical diagnosis	10	16.1
Biopsy diagnosis	52	83.9
Site of biopsy		
No biopsy	10	16.1
Thoracic lymph nodes	29	46.8
Extra thoracic lymph nodes	16	25.8
Skin	4	6.5
Other	3	4.8

### Smoking status

Smoking history was available for 72.6% of patients. Among patients with available data, 58.1% were non smokers, and 14.5% were current or former smokers. The remaining 27.4% of patients had no documented smoking status (Table 5).

### Comorbid conditions

Comorbidity data were available for 44 patients. Hypertension was the most frequently reported condition (24.2%), followed by diabetes mellitus (21%). Other less prevalent comorbidities included hypothyroidism (8.1%), gout (6.5%), chronic kidney disease (1.6%), and congestive heart failure (1.6%) (Table 5).

**Table 5:** Distribution of medical history of patients ($N=62$)

Medical history	n	%
Smoker		
Yes	9	14.5
No	36	58.1
Hypertension History		
Have	15	24.2
Haven’t	29	46.8
Diabetes Mellitus History		
Have	13	21.0
Haven’t	31	50.0
Gout History		
Have	4	6.5
Haven’t	40	64.5
Hypothyroidism History		
Have	5	8.1
Haven’t	39	62.9
Chronic Kidney Disease History		
Have	1	1.6
Haven’t	43	69.4
Congestive Heart Failure History		
Have	1	1.6
Haven’t	43	69.4

### Gender differences in smoking and comorbidities

Gender-based assessment of lifestyle and medical comorbidities revealed several notable patterns (Supplementary Table S1). The prevalence of smoking differed significantly between the sexes, with 20% of male patients reported tobacco use compared to 0% of female patients ($\chi ^{2} = 12.857$, $p = 0.0001$). Hypothyroidism also demonstrated a sex difference: it was present in 11.4% of females but in none of the males ($\chi ^{2} = 5.151$, $p = 0.023$).

Other comorbidities –including hypertension, diabetes mellitus, gout, chronic kidney disease, and congestive heart failure–did not exhibit statistically significant sex-based differences. These findings indicate differences in the distribution of comorbidities by sex within this cohort, particularly for smoking status and hypothyroidism.

### Age at diagnosis by gender

**Fig. 3:**
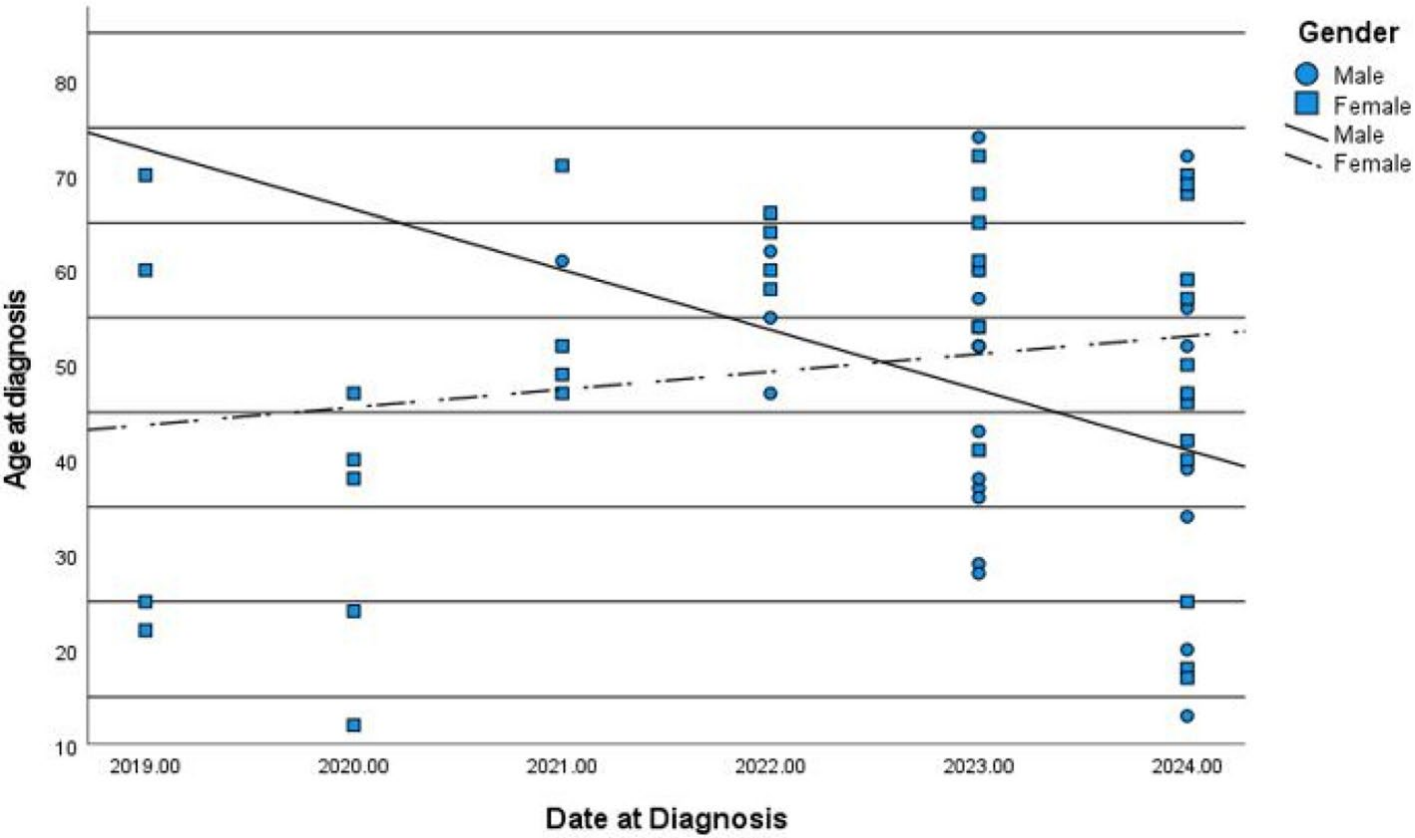
Trend in age at diagnosis stratified by sex, 2019–2024. This trend graph displays changes in mean age at diagnosis for male and female patients over the six-year period. While male patients trended toward younger age at diagnosis, female patients exhibited an increase in average diagnostic age, particularly in the later years

Exploratory analysis of age at diagnosis stratified by sex suggested differing temporal patterns (Fig. 3). Male patients showed an apparent decrease in age at diagnosis over time, with more recent cases occurring in younger age groups. Female patients appeared to show an increase in age at diagnosis in recent years,with the majority of new cases during 2023–2024 occurring among women in their 50 s and 60s.

A comparative analysis using Levene’s test and independent samples *t*-tests found no statistically significant difference in mean age at diagnosis between males and females (Supplementary Table S2). This was consistent regardless of variance assumptions:

- Equal variances assumed: $t = -0.722$, $p = 0.473$
- Equal variances not assumed: $t = -0.740$, $p = 0.463$

Mean age at diagnosis was 46.58 years (SD = 15.36) for males and 49.68 years (SD = 17.13) for females. Despite numerical differences, no statistically significant difference in mean age at diagnosis was observed between males and females, indicating comparable age distributions in this sample.

### Missing data analysis

A systematic assessment of data completeness revealed varying degrees of missing information in clinical and demographic variables (Supplementary Table S3). Missing data ware present in several variables, particularly smoking status and history of comorbidities, with missing data ranging from approximately 27.4% to 29%. This incompleteness reflects the retrospective design of the study and variability in historical medical record documentation. Because of the relatively small sample size and the risk of introducing additional bias through statistical imputation, missing values were not imputed. Instead, analyses involving variables with incomplete information were conducted using available-case (complete-case) data. The number of observations used for each analysis was therefore reported where applicable.

## Discussion

Sarcoidosis is a complex multisystem inflammatory disease characterized by noncaseating granuloma formation and can be challenging to diagnose, particularly in resource-limited settings [1, 7]. This study provides the first hospital-based epidemiological evaluation of sarcoidosis in the southern West Bank, Palestine, outlining incidence trends, demographic characteristics, diagnostic patterns, and comorbidity profiles over a six-year period. As such, it addresses an important gap in regional public health knowledge and contributes novel data from a previously underrepresented population.

A progressive increase in sarcoidosis incidence was observed over the study period, rising from 0.41 per 100,000 in 2019 to 1.92 per 100,000 in 2024, accompanied by a parallel increase in prevalence from 0.4 to 5.7 per 100,000. This increase in prevalence likely reflects cumulative case accumulation over time in addition to improved detection. Despite this rise, rates remain considerably lower than those reported in Northern Europe and North America, where large population-based studies and national registries have consistently demonstrated higher disease burden [1, 3, 5, 18–20]. These differences may be influenced by more comprehensive surveillance systems and greater access to advanced diagnostic modalities in high-income settings.

Similarly, sarcoidosis rates in Palestine were lower than those reported in several neighboring Middle Eastern countries. In Israel, hospital-based studies reported higher case detection [15], while regional cohorts from Kuwait highlighted greater clinical awareness and diagnostic capacity [21, 22]. Earlier studies from the region also documented variability in epidemiology, reflecting heterogeneity in study design and healthcare infrastructure [23].

Overall, regional variation in sarcoidosis epidemiology likely reflects differences in diagnostic capacity, access to imaging and biopsy techniques, population structure, and disease awareness rather than true biological differences alone. In Palestine, limited availability of specialized diagnostic tools and fragmented healthcare pathways may have contributed to underdiagnosis, particularly during the earlier years of the study

The apparent increase in sarcoidosis incidence observed after 2022 should be interpreted cautiously. Given the relatively small sample size and retrospective design, the observed rise may reflect improvements in case detection rather than a definitive increase in disease occurrence. Increased clinician awareness, expanded use of chest imaging following the COVID-19 pandemic, and higher referral rates to tertiary centers may have contributed to improved diagnostic recognition. Post-pandemic healthcare re-engagement may also have resulted in previously delayed cases being diagnosed. Although population aging and environmental exposures cannot be excluded as contributing factors, the present data are insufficient to confirm a true epidemiological increase. Future studies with larger sample sizes and more advanced trend analyses will be required to clarify temporal changes in sarcoidosis incidence [24, 25].

A clear female predominance was observed, consistent with findings from other Middle Eastern and Mediterranean populations [23, 26, 27]. The mean age at diagnosis aligns with regional reports [15]. Notably, nearly 33% of patients were diagnosed at 60 years or older, which may reflect delayed diagnosis or misattribution of symptoms to more prevalent chronic conditions in older adults [25]. Although females appeared to be diagnosed at older ages than males, this difference was not statistically significant and should be interpreted cautiously due to the limited sample size. Such patterns may reflect gender-related differences in healthcare utilization rather than intrinsic biological differences in disease onset.

Histopathological confirmation was achieved in the majority of cases, in line with international recommendations emphasizing tissue confirmation to exclude alternative causes of granulomatous disease such as tuberculosis and lymphoma [7, 8]. Thoracic lymph nodes were the most frequently sampled site, followed by extrathoracic lymph nodes and cutaneous tissue. Despite high biopsy confirmation rates, diagnostic challenges persist locally, including limited access to endobronchial ultrasound-guided biopsy (EBUS), inconsistent availability of high-resolution computed tomography, and overlap with endemic infections [25]. These barriers may delay diagnosis and contribute to underestimation of disease burden.

The comorbidity profile revealed substantial rates of hypertension and diabetes, consistent with previous studies linking sarcoidosis to chronic systemic inflammation, autoimmune susceptibility, and metabolic effects of long-term corticosteroid therapy [12–15]. Significant gender differences were observed, with smoking reported exclusively among males and hypothyroidism predominantly among females, reflecting known gender-specific risk patterns [26]. These factors may influence disease presentation and management; however, further investigation is required to determine their impact on long-term outcomes.

Although no geographic differences were detected within the study area, the southern West Bank encompasses heterogeneous environmental and occupational settings that may be relevant to the risk of sarcoidosis. The region includes extensive stone quarrying and construction activities, which may increase exposure to mineral dust and silica, as well as agricultural environments associated with organic particulates and bioaerosols. Such exposures have been implicated in granulomatous lung disease and have been proposed as potential environmental triggers of sarcoidosis [28]. Although environmental exposure data were not available in the present study, these regional conditions warrant consideration when interpreting disease patterns. Future studies in Palestine should incorporate detailed environmental and occupational exposure assessments to better evaluate possible environmental determinants of sarcoidosis in this population.

Clinical characterization was limited by the absence of Scadding radiographic staging, incomplete documentation of extrapulmonary involvement, and lack of treatment or follow-up information—gaps that restrict phenotype comparison with large international cohorts [6–8, 11]. Missing data—particularly for smoking status and comorbidities—were substantial and may not have been random, potentially leading to underestimation of certain associations. These challenges highlight the need for improved medical documentation and standardized data collection practices.

Beyond clinical manifestations, sarcoidosis carries important social and economic implications, particularly in resource-limited settings such as Palestine. Diagnostic delays may result in prolonged symptoms, reduced work productivity, and repeated healthcare visits. Long-term treatment with corticosteroids or immunosuppressive agents can impose financial strain on patients and healthcare systems, while indirect costs related to transportation, caregiving, and loss of income further compound disease burden. These impacts are especially relevant given the predominance of middle-aged and older adults in the cohort, many of whom manage multiple chronic conditions. Early diagnosis and coordinated care pathways are therefore critical to reducing both individual and societal costs.

Although detailed treatment data were not systematically available for analysis in this study, sarcoidosis management generally follows a stepwise approach based on ATS/ERS/WASOG recommendations. Asymptomatic or mildly symptomatic patients are often managed with observation, given the potential for spontaneous remission. Systemic corticosteroids remain first-line therapy for patients with clinically significant disease [29], with second-line immunosuppressive agents such as methotrexate or azathioprine recommended when corticosteroids are insufficient or poorly tolerated. For severe or refractory disease, biologic therapies, particularly anti-TNF agents, may be indicated. Regular follow-up, including pulmonary function testing, ophthalmologic assessment, and cardiac screening, is essential to detect disease progression and guide timely intervention [6]. This overview is provided for contextual understanding of disease burden rather than to evaluate treatment effectiveness.

The observed increase in sarcoidosis burden has important public health implications. Strengthening diagnostic capacity—particularly in imaging, interventional pulmonology, and pathology services—is essential. Increasing clinician awareness, especially in primary care and general medicine settings, may facilitate earlier recognition and referral [15]. Improving early detection and establishing structured referral pathways may help reduce diagnostic delays and improve patient outcomes. Given the frequency of associated chronic conditions, integrating sarcoidosis management into existing noncommunicable disease frameworks may improve continuity of care. Establishing a national registry for sarcoidosis would improve surveillance, enable multicenter collaboration, and support robust epidemiological and clinical research.

Several limitations should be acknowledged. The retrospective, hospital-based design limits generalizability and may underestimate true disease burden by excluding mild or asymptomatic cases. Reliance on a single referral center, substantial missing data, lack of environmental exposure assessment, and limited clinical detail may introduce bias and restrict interpretability. These limitations should be carefully considered when comparing the present findings with other populations.

Future studies should adopt prospective, multicenter designs with standardized clinical phenotyping, including radiographic staging, pulmonary function testing, and systematic assessment of extrapulmonary involvement. Incorporation of environmental, occupational and genetic exposure data along with longitudinal follow-up—will be essential to clarify disease determinants, evaluate treatment results, and inform evidence-based health policy in Palestine and similar resource-limited settings.

## Limitations

This study has several limitations that should be considered when interpreting the findings. First, its retrospective hospital-based design may introduce selection bias, as cases diagnosed in private clinics or undiagnosed community cases could be underrepresented, potentially leading to an underestimation of the true disease burden. Although Al-Ahli Hospital serves as the primary tertiary referral center for histopathological confirmation of sarcoidosis in the southern West Bank, some patients—particularly those with mild disease managed clinically without biopsy or those referred outside the region—may not have been captured in the hospital registry. Additionally, asymptomatic or mildly symptomatic individuals who did not seek hospital care are likely to be missed, further affecting case ascertainment.

Second, the relatively small sample size of 62 patients over six years limits statistical power and restricts the ability to perform robust inferential analyses, which may affect the generalizability of the findings. Third, some clinical and demographic data were incomplete, including missing smoking status in approximately 28% of patients and incomplete medical history records for a portion of the cohort. In addition, incomplete and non-standardized documentation of clinical details—particularly radiographic staging (e.g., Scadding classification), presenting symptoms, and extrapulmonary organ involvement—limited detailed clinical phenotyping and restricted meaningful comparisons with larger international sarcoidosis cohorts.

Finally, the study did not include detailed information on treatment patterns, clinical outcomes, environmental exposures, or genetic factors that may influence disease presentation and progression. These factors may be important for understanding regional variations in sarcoidosis.

Despite these limitations, this study represents an important first step in characterizing sarcoidosis in Palestine. It provides baseline epidemiological and clinical data and highlights the need for future multicenter, prospective, and population-based studies incorporating standardized clinical documentation, as well as environmental and genetic data to better understand disease determinants and support evidence-based public health planning.

## Conclusion

Sarcoidosis remains an uncommon yet clinically significant disease in southern Palestine, and predominantly affects middle-aged adults with a slight female predominance. Over the study period (2019–2024), both the incidence and prevalence of sarcoidosis showed an upward trend, which may reflect improved disease recognition and diagnostic awareness, although a true epidemiological increase cannot be excluded.

This study establishes essential baseline epidemiological data for sarcoidosis in Palestine and underscores the need for a strengthened public health response. Strengthening diagnostic capacity, enhancing clinician education, and expanding access to histopathology and advanced imaging services are critical for early detection and optimal patient management. Future multicenter and prospective studies should explore environmental, occupational, and genetic risk factors to refine clinical characterization, assess treatment outcomes, and guide evidence-based health policies in resource-limited settings.

## Supplementary Information

Supplementary Material 1.

## Data Availability

The datasets generated and/or analyzed during the current study are available from the corresponding author on reasonable request.

## Abbreviations

CT: Computed Tomography
IRB: Institutional Review Board
PCBS: Palestinian Central Bureau of Statistics
PRCS: Palestine Red Crescent Society
SPSS: Statistical Package for the Social Sciences
TNF-$\alpha$: Tumor Necrosis Factor-alpha
VATS: Video-Assisted Thoracoscopic Surgery
WASOG: World Association for Sarcoidosis and Other Granulomatous Disorders
