# Supplementary material for: Epidemiology and demographic trends of sarcoidosis in southern West Bank, Palestine: a retrospective study (2019–2024)
Source: BMC Public Health. 2026 May 22;26:2149. doi: 10.1186/s12889-026-27878-0 (PMC13371677; doi:10.1186/s12889-026-27878-0)
Supplement: Supplementary file 1 — Supplementary Material 1. [file 12889_2026_27878_MOESM1_ESM.pdf]

Supplementary

Supplementary Table S1: Gender differences according smoking and co-morbidities of cases

Health issue	Variable	Gender		Chi-square statistic	P-value
		Male	Female		
Smoking	Yes	9 (20%)	0 (0.0%)	12.857	.0001
	No	12 (26.7%)	24 (53.3%)		
Hypertension	Have	5 (11.4%)	10 (22.7%)	1.890	.169
	Haven't	16 (36.4%)	13 (29.5%)		
Diabetes Mellitus	Have	4 (9.1%)	9 (20.5%)	2.127	.145
	Haven't	17 (38.6%)	14 (31.8%)		
Gout	Have	3 (6.8%)	1 (2.3%)	1.312	.252
	Haven't	18 (40.9%)	22 (50.0%)		
Hypothyroidism	Have	0 (0.0%)	5 (11.4%)	5.151	.023
	Haven't	21 (47.7%)	18 (40.9%)		
Chronic kidney disease	Have	0 (0.0%)	1 (2.3%)	.934	.334
	Haven't	21 (47.7%)	22 (50.0%)		
Congestive Heart Failure	Have	1 (2.3%)	0 (0.0%)	1.121	.290
	Haven't	20 (45.5%)	23 (52.3%)		

Supplementary Table S2: Comparison of Age at Diagnosis by Gender Using Levene's Test and t-Test

		*t-test for Equality of Means						
		t	df	Sig. (2-tailed)	Mean Difference	Std. Error Difference	95% Confidence Interval of the Difference	
							Lower	Upper
Age at diagnosis (years)	Equal variances assumed	-.722	60	.473	-3.101	4.296	-11.694	5.492
	Equal variances not assumed	-.740	52.989	.463	-3.101	4.190	-11.506	5.304

Note. Mean for male= 46.58 with SD 15.36, Mean for female=49.68 with SD 17.131

Supplementary Table S3: Distribution of missing data entry (n=62)

Variables	n	%
**Smoker**	17	27.4
**Hypertension History**	18	29
**Diabetes Mellitus History**	18	29
**Gout History**	18	29
**Hypothyroidism History**	18	29
**Chronic Kidney Disease History**	18	29
**Congestive Heart Failure History**	18	29

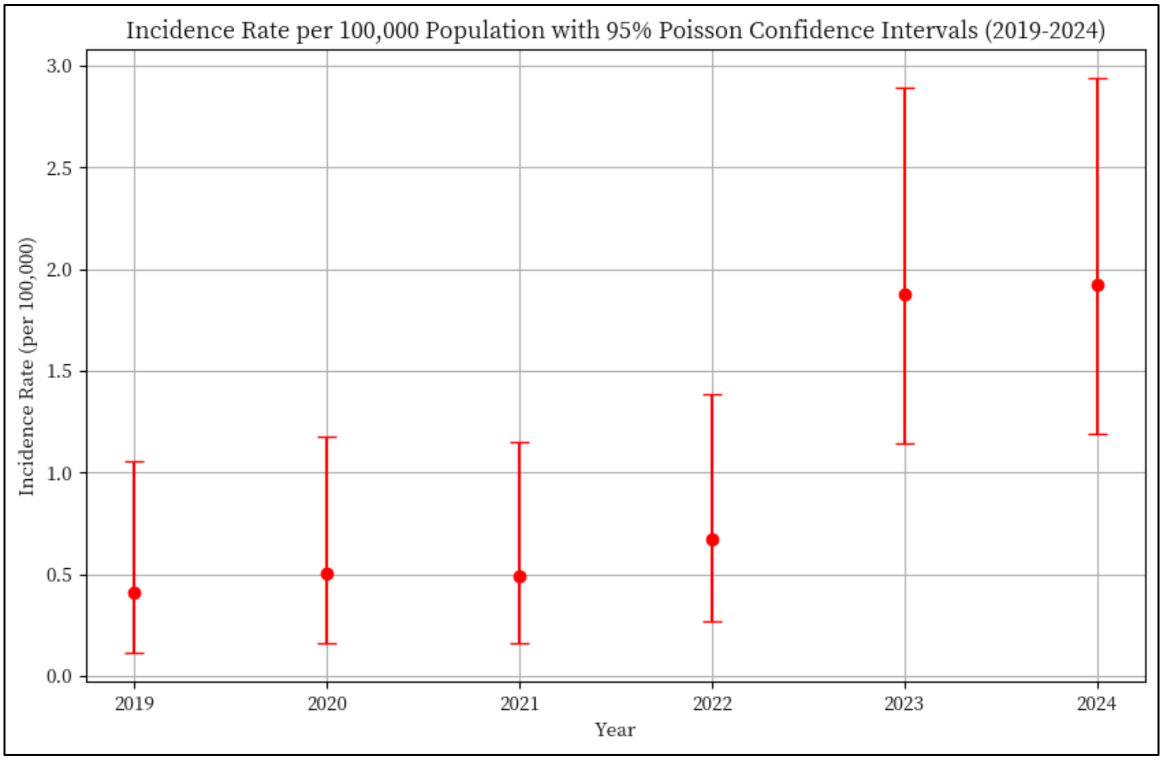

Supplementary Figure S1: Incidence rate of Sarcoidosis for Hebron and Bethlehem over 2019–2024 with 95% Poisson Confidence Intervals. *This figure illustrates the annual incidence rates per 100,000 populations along with their corresponding 95% Poisson confidence intervals. A relatively stable pattern is observed between 2019 and 2022, followed by a pronounced increase in 2023 and 2024. The widening upper confidence bounds in these latter years reflect the substantial rise in newly reported cases during this period*

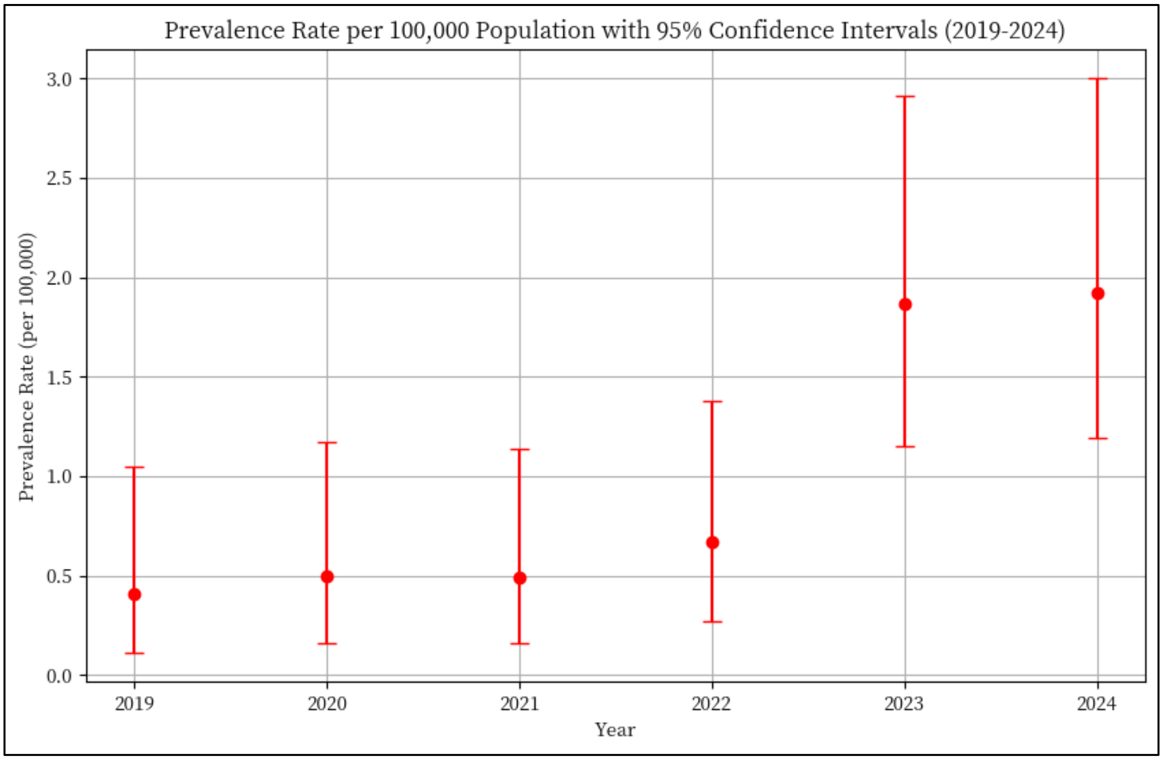

Supplementary Figure S2: Prevalence rate of sarcoidosis with 95% confidence intervals in Hebron and Bethlehem, 2019–2024. *This figure illustrates the annual prevalence rate of sarcoidosis per 100,000 population across the study period, accompanied by 95% confidence intervals. A steady increase is observed from 2019 through 2022, followed by a sharp rise in 2023 and 2024. The widening intervals in the later years indicate increased variability associated with higher case numbers and population adjustments.*
